# Targeting heat shock proteins 90 and 70: A promising remedy for both autoimmune bullous diseases and COVID-19

**DOI:** 10.3389/fimmu.2022.1080786

**Published:** 2022-12-15

**Authors:** Michael Kasperkiewicz, Stefan Tukaj

**Affiliations:** ^1^ Department of Dermatology, Keck School of Medicine, University of Southern California, Los Angeles, CA, United States; ^2^ Department of Molecular Biology, Faculty of Biology, University of Gdańsk, Gdańsk, Poland

**Keywords:** autoimmune bullous disorders (AIBDs), heat shock proteins (HSPs), Hsp90, Hsp70, COVID-19, SARS-CoV-2

## Abstract

Heat shock proteins (Hsps), including Hsp90 and Hsp70, are intra- and extracellular molecules implicated in cellular homeostasis and immune processes and are induced by cell stress such as inflammation and infection. Autoimmune bullous disorders (AIBDs) and COVID-19 represent potentially life-threatening inflammatory and infectious diseases, respectively. A significant portion of AIBDs remain refractory to currently available immunosuppressive therapies, which may represent a risk factor for COVID-19, and suffer from treatment side-effects. Despite advances in vaccination, there is still a need to develop new therapeutic approaches targeting SARS-CoV-2, especially considering vaccine hesitancy, logistical distribution challenges, and breakthrough infections. In this mini review, we briefly summarize the role of targeting Hsp90/70 as a promising double-edged sword in the therapy of AIBDs and COVID-19.

## Introduction

The expression of heat shock proteins (Hsps), including Hsp90 and Hsp70, can be up-regulated by various stress factors including inflammation and infection. As chaperones, they classically interact with protein substrates and (co-)chaperones to maintain cellular homeostasis by participating in correct protein folding or stability, as well as cell differentiation, survival, and death. In addition, both intra- and extracellular Hsps have an integral role in inflammatory responses through active involvement in a wide range of immune processes (1).

Autoimmune bullous diseases (AIBDs), comprising pemphigus and pemphigoid, are potentially life-threatening blistering disorders of the skin and/or mucosa mediated by autoantibodies against desmosomal and hemidesmosomal structures, respectively. Patients usually require long-term potent immunosuppressive treatments including systemic corticosteroids and adjuvant immunomodulators. A significant portion of cases remain refractory to currently available therapies and suffer from treatment side-effects (2, 3).

COVID-19 is a disease caused by the novel coronavirus SARS-CoV-2 which has led to a global devastating pandemic since March 2020. The clinical presentation ranges from mild or even asymptomatic courses to critical symptoms such as respiratory failure and multi-organ dysfunction. Despite advances in vaccination, there is still a need to develop new therapeutic approaches targeting SARS-CoV-2, especially considering vaccine hesitancy, logistical distribution challenges, and breakthrough infections (4).

Patients with AIBDs have faced considerable challenges during the COVID-19 outbreak, taking into account that their immunosuppressive treatments, particularly the B-cell depleting agent rituximab (a first-line drug approved for pemphigus), may predispose them to more severe COVID-19 and compromise vaccine immunogenicity (5, 6). In addition, there have been some reports on SARS-CoV-2 vaccine-induced/exacerbated AIBD cases (7). Therefore, an ideal treatment during this pandemic would be a new medication that covers both AIBDs and COVID-19.

In this mini review, which is dedicated to the memory of our honourable mentor Professor Detlef Zillikens, we briefly summarize the role of targeting Hsp90/70 as a promising double-edged sword in the therapy of AIBDs and COVID-19.

## Hsp90/70 and AIBDs

Several lines of evidence suggest that Hsp90 is involved as a notable pathophysiological factor in AIBDs including epidermolysis bullosa acquisita (EBA) and bullous pemphigoid. The anti-inflammatory clinical and molecular effects of anti-Hsp90 treatment were mostly demonstrated in several EBA studies using corresponding *in vivo* mouse models, *ex vivo* human dermal-epidermal separation cryosection experiments, and *in vitro* rodent and human cell culture assays (8, 9). Hsp90 inhibitors, including 17-DMAG, 17-AAG, and TCBL-145, exhibited activity by potently affecting inflammatory disease pathways (e.g., suppression of effector T-cells, B-cells, and neutrophils; down-regulation of NF-κB activity; blunting of autoantibody, pro-inflammatory cytokine, and reactive oxygen species production; inhibition of matrix metalloproteinases; promotion of regulatory B cells) (10–13). With regards to bullous pemphigoid, human skin biopsy analyses as well as human cell culture and serology assays revealed that Hsp90 is aberrantly expressed and secreted in these patients and that its blockade modulates autoantibody-induced IL-8 production by cultured keratinocytes (14, 15).

Compared to Hsp90, the role of Hsp70 in AIBDs has been overall less studied (9). Two recent studies indicated that both extracellular Hsp70 and autoantibodies to Hsp70 display pro-inflammatory activities in the context of EBA development (16, 17). In the first study, the *in vivo* pathophysiological relevance of extracellular Hsp70 was demonstrated in mice with experimental EBA in which elevated blood levels of this chaperone were recorded. Hsp70-treated animals had a more pronounced clinical disease severity compared to controls which was paralleled by increased levels of cutaneous matrix metalloproteinase 9 and plasma hydrogen peroxide, with the latter observation being confirmed in an independent EBA-specific reactive oxygen species release assay. In addition, cell culture experiments using human naive peripheral blood mononuclear cells revealed that extracellular Hsp70 stimulated the secretion of the T-cell-derived pro-inflammatory cytokines IL-6 and IL-8 (16). In the second study, it was demonstrated that autoantibodies to Hsp70 may contribute to EBA development *via* enhanced neutrophil infiltration to the skin and activation of the NF-κB signalling pathway in an IFN-γ-associated manner (17).

## Hsp90/70 and COVID-19

To initiate infection, SARS-CoV-2 enters the human host cell *via* binding of its spike protein to cell surface receptors including angiotensin-converting enzyme 2, followed by endocytosis or fusion with the surface membrane, viral gene transcription, translation, and replication (4). Of note, SARS-CoV-2 is dependent on host molecular chaperones, mainly Hsp90/70, to accomplish these entries and/or replication steps (18, 19).

Hsp90 has been shown to be over-expressed in the damaged lungs of COVID-19 subjects (20), and Hsp90 inhibitors (AT13387 and AUY-922) were able to prevent and repair SARS-CoV-2 spike protein-induced pulmonary microvascular endothelial dysfunction (21). Wyler et al. demonstrated that inhibition of Hsp90 activity by onalespib, ganetespib, or 17-AAG resulted in a reduction of both SARS-CoV-2 replication and expression of pro-inflammatory cytokines in primary human airway epithelial cells (22). Similar results were obtained by Goswami et al. using the Hsp90 inhibitor SNX-5422 (23). In addition, *in vitro* experiments revealed inhibition of SARS-CoV-2 replication by the Hsp90 inhibitors 17-AAG and tanespimycin, respectively (24, 25). Lowering of SARS-CoV-2 propagation by pharmacological Hsp90 inhibition was confirmed by a computational study of patient RNA sequencing data (26).

Two members of the Hsp70 family, HSPA1L and GRP78, have been implicated in the modulation of SARS-CoV-2 (27–30). It has been suggested that SARS-CoV-2-infected cells epigenetically up-regulate HSPA1L, leading to the over-production of Hsp70 to facilitate SARS-CoV-2 replication in host cells (27). In addition, computer modelling predictions and experimental studies have shown that GRP78 assists in the host cell recognition of SARS-CoV-2 spikes and viral entry (28–30).

## Role of molecular mimicry

A link between COVID-19 and the development of autoimmunity has been suggested, which is based on the assumption that molecular mimicry exists between immunogenic proteins of SARS-CoV-2 and human molecules including Hsps and autoantigens of AIBDs (31). This hypothesis has been recently disproven by two studies showing that autoantibodies to Hsp90/70/60 are not altered in anti-SARS-CoV-2 IgG-seropositive humans and that these circulating anti-SARS-CoV-2 antibodies do not cross-react with pemphigus or pemphigoid autoantigens (32, 33).

## Conclusions

**Figure 1:**
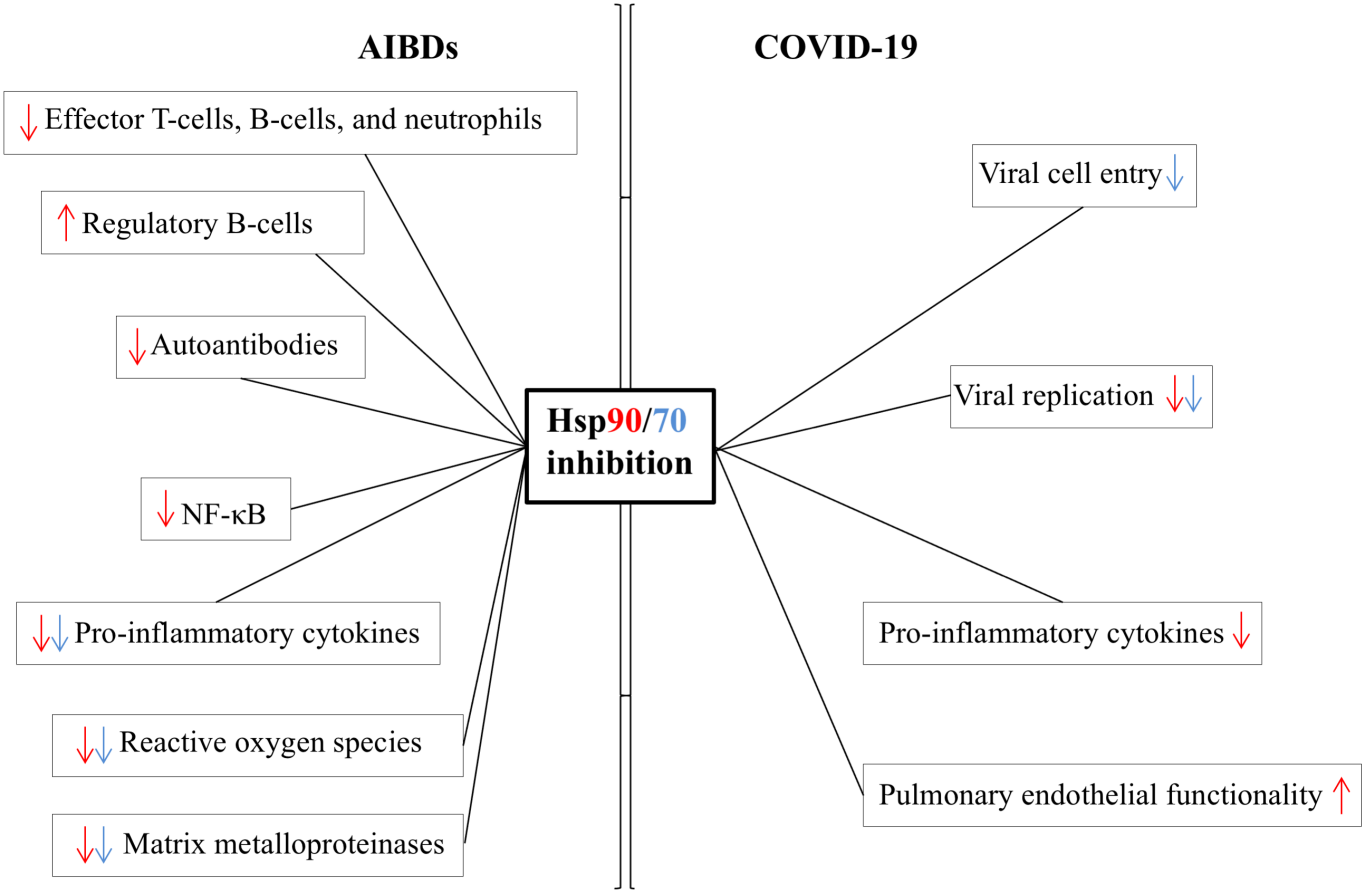
Proposed mode of action of targeting Hsp90/70 in AIBDs and COVID-19 based on current scientific evidence. AIBD data are derived from studies on epidermolysis bullosa acquisita and bullous pemphigoid. Although Hsp70 has been involved in the depicted pathophysiological factors/processes of AIBDs and COVID-19, data on the effects of true Hsp70 inhibition are limited to experiments on SARS-CoV-2 cell entry so far and remain speculative otherwise. The red and blue colours of arrows correspond to Hsp90 and Hsp70, respectively.

Hsps90/70 are involved in AIBDs and COVID-19 in many different ways and, thus, can be important therapeutic targets for both conditions, an ideal scenario during this pandemic (**Figure 1**). However, several questions remain open. Independent reports on the role of Hsps90/70 in either AIBDs or COVID-19 do not ultimately imply a direct correlation between these stress proteins and both diseases, which is hampered by the fact that all information is limited to preclinical studies so far (9, 18, 19). In addition, the family of Hsps is large, and the interaction between each other and their client proteins is complex (1). Thus, targeting one of the Hsps may lead to a ripple effect. As an example, inhibition of Hsp90 classically results in the activation of heat shock factor 1 and, consecutively, the over-expression of intracellular Hsp70. In contrast to extracellular Hsp70, intracellular Hsp70 has mostly anti-inflammatory properties by blocking NF-κB activation, a desirable effect for both AIBDs and COVID-19 (i.e., cytokine storm) (34). On the other hand, over-production of intracellular Hsp70 could potentially facilitate SARS-CoV-2 replication. Therefore, further exploration of the net effects of especially clinically available Hsp90 blockers in basic studies and clinical trials is needed.

## Author contributions

MK drafted and ST edited the manuscript. All authors contributed to the article and approved the submitted version.
